# Performance Evaluation of Warm-Mix Agents on Crumb Rubber-Modified Asphalt

**DOI:** 10.3390/ma19112333

**Published:** 2026-06-01

**Authors:** Bo Huang, Song Xu, Shishui Liulin, Xiangjie Niu, Jihong Zhou, Xiong Xu

**Affiliations:** 1 Fujian Transportation Development High-Tech Co., Ltd., Fuzhou 350004, China; 2 College of Civil Engineering, Fuzhou University, Fuzhou 350108, China; 3 School of Civil Engineering and Architecture, Wuhan Institute of Technology, Wuhan 430073, China; xxucea@wit.edu.cn

**Keywords:** crumb rubber-modified asphalt, warm-mix technology, warm-mix agents, rheological properties, performance evaluation

## Abstract

To achieve warm-mix production of crumb rubber-modified asphalt (CRA), an organic warm-mix agent, a surfactant-based warm-mix agent, and a composite warm-mix agent were employed to prepare warm-mix CRA. The effects of warm-mix agents on the physical properties of CRA were evaluated using the penetration test, softening point test, viscosity test, ductility test, and elastic recovery test. The effects of warm-mix agents on the high- and low-temperature rheological properties were investigated through dynamic shear rheometer (DSR), multiple stress creep recovery (MSCR), and bending beam rheometer (BBR) tests. Moreover, the viscosity–temperature characteristics and the VOC emissions of different warm-mix CRAs were explored. The results show that Sasobit, an organic warm-mix agent, increases the elastic fraction and stiffness of CRA, which enhances its high-temperature resistance to permanent deformation but compromises its low-temperature cracking resistance. UWM, a surfactant-based warm-mix agent, elevates the viscous fraction and flexibility of CRA, which improves its low-temperature cracking resistance but weakens its high-temperature rutting resistance. The composite warm-mix agent, consisting of 2 wt.% Sasobit and 5 wt.% UWM, can balance the stiffness and flexibility of CRA, endowing CRA with satisfactory pavement performance. All three warm-mix agents effectively reduce the viscosity, mixing temperature, and VOC emissions of CRA. The composite warm-mix agent reduces the VOC emissions of CRA by 53.0%, exhibiting the most pronounced reduction.

## 1. Introduction

Crumb rubber-modified asphalt (CRA) has been widely used in pavement construction due to its excellent pavement performance, low construction cost, and favorable environmental effects [1,2,3]. However, when the crumb rubber dosage is high, the viscosity of CRA increases markedly, which results in higher production, mixing, and compaction temperatures [4,5,6]. The elevated production and paving temperatures consequently lead to increased emissions of volatile organic compounds (VOCs) from CRA.

In order to reduce the production and construction temperatures of CRA and mitigate its VOC emissions, warm-mix technology has attracted considerable attention [7,8,9]. The commonly used warm-mix technologies at present mainly include foaming-based, organic additive-based, and surfactant-based methods. The foaming-based method involves the rapid vaporization of water within hot asphalt, which generates numerous microbubbles that temporarily reduce the viscosity of asphalt. This method enhances the workability of asphalt and asphalt mixtures, but it adversely affects the adhesion between asphalt and aggregates [10,11]. In addition, it requires specialized foaming equipment and control systems, which increase capital investment and construction management costs and compromise the economic feasibility in some projects [12]. Compared with the foaming-based method, the organic additive-based and surfactant-based methods do not require specialized equipment and, therefore, offer more satisfactory economic feasibility. Sasobit, whose main component is long-chain hydrocarbon wax, is considered the most effective organic additive for the reduction in asphalt viscosity and the lowering of its mixing and compaction temperatures [13,14]. Under high-temperature conditions, Sasobit melts and forms a uniformly dispersed microcrystalline network within the binder, which improves the flowability and workability of asphalt and enhances the high-temperature rutting resistance of asphalt [15,16]. However, Sasobit recrystallizes at low temperatures, which increases the stiffness of the binder and, consequently, reduces the low-temperature cracking resistance of asphalt. For instance, A. Amini et al. [17] found that the addition of Sasobit improves the high-temperature performance and fatigue performance of the binder. Zhao et al. [18] reported that Sasobit weakens the low-temperature performance of asphalt, and its dosage should not exceed 3 wt.% by weight of asphalt in cold regions. Zhang et al. [19] investigated the effect of Sasobit on the microstructure of asphalt using molecular simulation and found that the addition of Sasobit enhances the packing density of asphaltenes and the cohesive energy density of asphalt, which reduces the low-temperature crack resistance of asphalt. Zhang et al. [20] found that wax-based warm-mix agents with long carbon chains exhibit poor compatibility with asphalt, whereas the incorporation of ethylene-vinyl acetate polymers can effectively alleviate this issue. The surfactant-based method is also considered an effective method to reduce the high-temperature viscosity of asphalt and improve its workability. The addition of surfactant agents can change the interfacial tension and wettability between asphalt and aggregates, which enhances the workability of asphalt mixtures [21]. The representative surfactant agents include Evotherm and Cecabase RT. Evotherm belongs to the class of cationic quaternary ammonium surfactants [22]. Cecabase RT is a cationic surfactant-based warm-mix additive [23]. Notably, the relatively high cost of surfactant agents limits their widespread application. Moreover, the addition of surfactant agents weakens the high-temperature rheological properties of asphalt [24,25].

As mentioned above, the high viscosity of CRA often necessitates elevated mixing and compaction temperatures to ensure adequate workability during construction, resulting in significant VOC emissions and raising environmental concerns. The application of warm-mix technology provides an effective means to reduce the viscosity of CRA and suppress the VOC emissions of CRA, which contributes to environmentally friendly and sustainable pavement construction. However, different warm-mix additives affect asphalt in distinct ways, and it is essential to lower the mixing and compaction temperatures without compromising pavement performance.

In this work, the effects of warm-mix agents on the physical and rheological properties of CRA were investigated through physical and rheological tests. The effects of warm-mix agents on the chemical properties of CRA were explored using Fourier-transform infrared spectroscopy. The effects of warm-mix agents on the VOC emissions from CRA were analyzed using gas chromatography–mass spectrometry (GC-MS). Furthermore, a composite warm-mix agent, consisting of an organic agent and a surfactant agent, was proposed to enhance warm-mix effectiveness, reduce VOC emissions, and improve the high- and low-temperature performance of CRA.

## 2. Materials and Methods

### 2.1. Materials

Petroleum asphalt 70#, 60-mesh crumb rubber, aromatic oil, and sulfur were used to prepare crumb rubber-modified asphalt (CRA). Crumb rubber was produced from waste tires via mechanical processing and supplied by Huayi Rubber Co., Ltd. (Shanghai, China). An organic warm-mix agent, a surfactant-based warm-mix agent, and a composite warm-mix agent were employed in this work. The commercial product, Sasobit, was used as the organic warm-mix agent and was supplied by Sasol Wax (Hamburg, Germany). The warm-mix agent UWM was selected as the surfactant-based warm-mix agent and was supplied by Fujian Transportation Development High-Tech Co., Ltd. (Fuzhou, China). The main components of UWM include surfactants, resins, and others. The composite warm-mix agent consisted of Sasobit and UWM. The main properties of the materials used are summarized in Table 1.

### 2.2. Preparation of Crumb Rubber-Modified Asphalt

The dosages of materials and preparation parameters were determined based on our preliminary exploratory experiments and previous studies [26,27,28]. Aromatic oil at 5 wt.% was added to the base asphalt and stirred for 10 min at 220 °C. Crumb rubber at 30 wt.% and 0.3% sulfur were added to the above mixture and mixed at 800 rpm for 60 min to ensure preliminary blending. The mixture was then subjected to high-shear treatment at 3000 rpm and 220 °C for 90 min, which promotes the swelling and degradation of crumb rubber. After shearing, the crumb rubber-modified asphalt was placed in an oven at 190 °C for 60 min. The above dosages were calculated based on the mass of the base asphalt.

### 2.3. Preparation of Warm-Mix Crumb Rubber-Modified Asphalt

**Figure 1:**
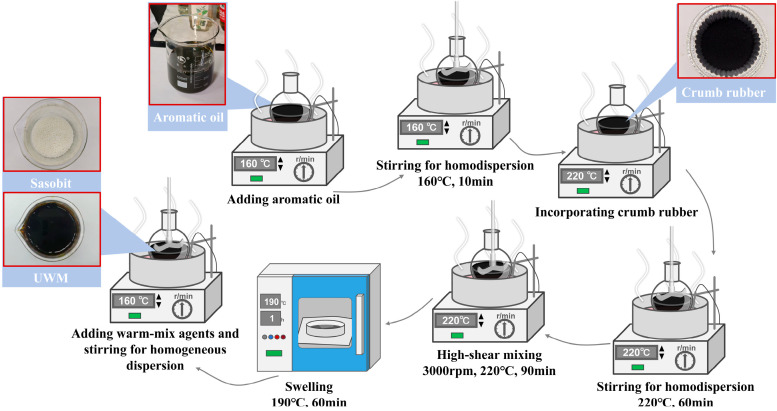
Detailed preparation procedure of warm-mix crumb rubber-modified asphalt.

The dosages of warm-mix agents were determined based on our preliminary exploratory experiments and our previous work [29]. The prepared crumb rubber-modified asphalt (CRA) was heated to 160 °C, and then the warm-mix agents (UWM and Sasobit) were added to the binder and stirred at 800 rpm for 30 min. The dosages of the warm-mix agents, expressed by weight of CRA, were selected as listed in Table 2. The detailed preparation procedure is shown in Figure 1. Moreover, the dosage of each component in the composite system was also calculated independently based on the mass of CRA.

### 2.4. Physical and Rheological Property Tests

#### 2.4.1. Physical Property Test

The physical properties of the binder samples were evaluated as per JTG E20-2011 [30], which includes penetration, ductility, the softening point, viscosity and the elastic recovery rate, respectively. Moreover, the viscosity values of all binders were analyzed at five temperatures (135 °C, 145 °C, 150 °C, 165 °C, and 180 °C), and the obtained data were fitted with the Saal model to predict the viscosity of CRA. The Saal model is presented in Equation (1). The viscosity test was conducted using a Brookfield rotational viscometer equipped with spindle No. 28, while the torque was maintained within the effective range of 10–90%, in accordance with JTG E20-2011.

(1) lglg(η×1000)=n−mlg(T+273.13)

where *η* is the viscosity of the binder (Pa·s), *T* is the test temperature (°C), and *n* and *m* are the fitting coefficients.

#### 2.4.2. High-Temperature Rheological Property Test

The high-temperature rheological parameters, including the complex modulus (G*), phase angle (δ), rutting factor (G∗/sinδ), percent recovery (R), and non-recoverable creep compliance (Jnr), obtained from temperature sweep (TS) and multiple stress creep recovery (MSCR) tests, were used to evaluate the high-temperature rheological properties of the binder samples. The TS and MSCR tests were conducted using a dynamic shear rheometer (DSR, MCR102, Anton Paar, Graz, Austria) at an oscillation frequency of 1.5 Hz, in accordance with JTG E20-2011. The average value of three replicate samples was reported. The detailed testing conditions for the TS and MSCR tests are summarized in Table 3.

#### 2.4.3. Low-Temperature Rheological Property Test

The low-temperature rheological parameters, including creep stiffness (S) and creep rate (m-value), obtained from the bending beam rheometer (BBR) test were used to evaluate the low-temperature rheological properties of the binder samples. The BBR test was conducted using a bending beam rheometer (BBR, Cannon SM-102, Huntington, NY, USA). A constant load of 980 mN was applied, and the test temperatures were set at −12 °C, −18 °C, and −24 °C. The details were conducted as per JTG E20-2011.

### 2.5. Fourier-Transform Infrared Spectroscopy Test

Fourier-transform infrared spectroscopy (FTIR) was used to investigate the modification effect of Sasobit and UWM on the CRA. A total of 0.2 g of the binder was dissolved in 4 mL of carbon disulfide (CS_2_) to prepare the asphalt solution. Two KBr pellets with a transmittance of about 90% were used, with one serving as the blank reference and the other used for the sample preparation. Then, 75 μL of the asphalt solution was dropped onto the center of the KBr pellet using a pipette. The spectra were collected over the wavenumber range of 4000–500 cm^−1^, with a resolution of 4 cm^−1^ and 32 scans.

### 2.6. VOC Emissions Test

#### 2.6.1. VOC Collection

In our previous research [31], a self-made VOC collection device was used to analyze the VOC emissions. The self-made device is mainly composed of a vacuum pump, a three-necked flask in an oil bath, a stirring unit and a cyclohexane trapping bottle. The binder samples were added to a three-necked flask and heated to the designated mixing temperature. VOCs emitted from the binder samples were captured by cyclohexane. The VOC collection process lasted for 30 min, during which VOCs were collected every 2 min, with each collection lasting 1 min.

#### 2.6.2. Gas Chromatography–Mass Spectrometry Test

A gas chromatograph–mass spectrometer (GCMS-QP2020 NX, Shimadzu, Kyoto, Japan) was used to analyze the chemical components of the VOC samples. The details of the GC-MS test are shown in Table 4 and Table 5. The specific procedures were conducted according to the literature [31].

The concentration of VOCs was quantified using the widely recognized external standard method, as reported in the literature [32]. Toluene solutions at different concentrations were prepared as standards to analyze the relationship between the chromatographic peak areas of VOC samples obtained from the GC–MS test and their concentrations, as described in Equation (2). Notably, a VOC quantification calibration curve was constructed using five concentrations of toluene standard solutions, as shown in Table 6. The calibration curve exhibited an R^2^ greater than 0.99, indicating excellent linearity over the tested concentration range. The detailed calculation procedure followed our previous research [33].

(2) $${VOC}_{x}=S_{x}\times \frac{{VOC}_{t}}{S_{A}}$$

where *VOC_x_* is the concentration of the analyte sample (mg/m^3^), *S_x_* is the peak area of the analyte sample, *VOC_t_* is the concentration of the standard (mg/m^3^), and *S_A_* is the peak area of the standard.

## 3. Results and Discussion

### 3.1. Physical Properties

**Figure 2:**
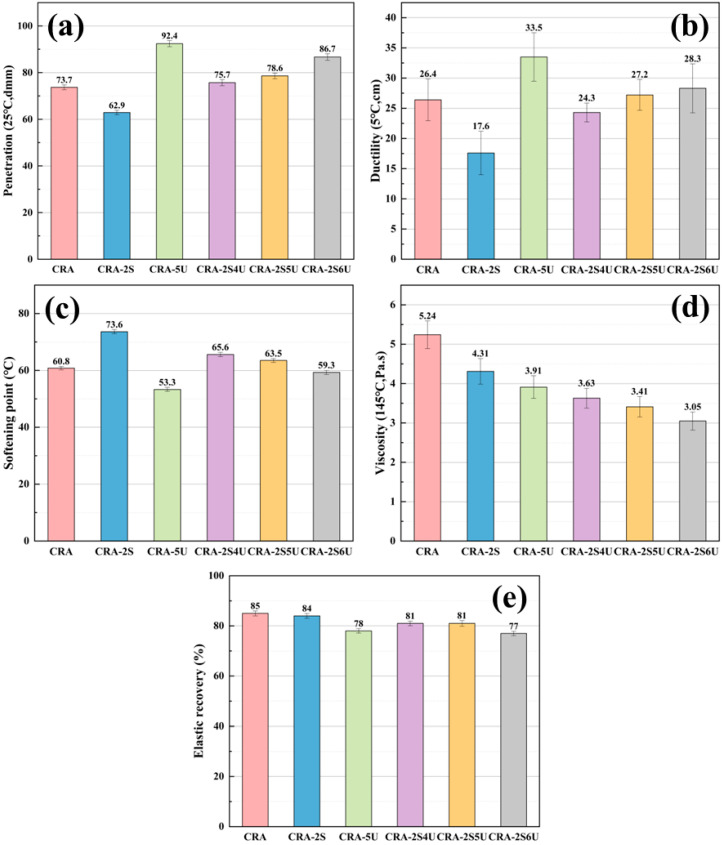
Physical properties of all binders: (**a**) penetration, (**b**) ductility, (**c**) softening point, (**d**) viscosity and (**e**) elastic recovery.

Figure 2 shows the physical properties of all binders. As observed, compared with CRA, the penetration and ductility of CRA-2S decreased by 14.7% and 33.3%, respectively, while the softening point increased by 21.1%, which indicates that the addition of Sasobit enhances the stiffness of CRA and its resistance to flow. Compared with CRA-2S, the penetration and ductility of CRA-2S4U, CRA-2S5U, and CRA-2S6U showed an increasing trend, while the softening point values of these samples continuously decreased. This change implies that the incorporation of UWM improves flowability and low-temperature ductility. Moreover, the penetration and ductility of CRA-5U increased by 25.4% and 26.9% relative to CRA, while the softening point decreased by 12.3%, which also indicates that the addition of UWM improves the low-temperature flexibility of the binder and weakens high-temperature performance. As illustrated in Figure 2e, the addition of Sasobit exerted a negligible influence on the elastic recovery property, which is considered favorable. However, the elastic recovery of CRA-5U decreased by 8.2% compared with CRA, which implies that UWM weakens the elastic recovery capacity of the binder. According to JTG F40-2004 [34], the elastic recovery of polymer-modified asphalt should exceed 75%, and the softening point should be higher than 60 °C. The elastic recovery and softening point of CRA-2S4U and CRA-2S5U remained above 75% and 60 °C, respectively. However, the softening point of CRA-2S6U fell below 60 °C. Given that warm-mix agents are intended to reduce the viscosity of CRA, CRA-2S5U exhibited favorable high- and low-temperature performance and elastic recovery, while maintaining a relatively low viscosity. Consequently, the composite warm-mix agent consisting of 2 wt.% Sasobit and 5 wt.% UWM is recommended.

### 3.2. Viscosity–Temperature Characteristics

**Figure 3:**
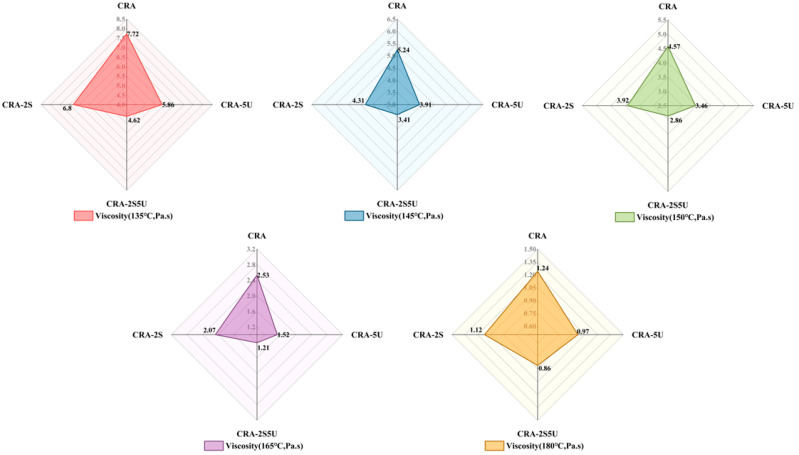
Viscosity values of all binders at different temperatures.

**Figure 4:**
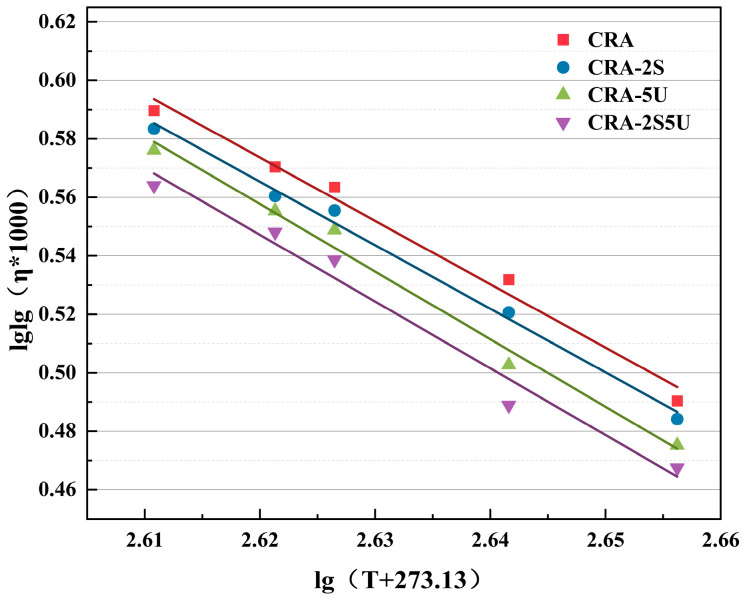
Viscosity–temperature curves of all binders.

The viscosity of CRA serves as a crucial parameter for determining the appropriate mixing and compaction temperatures of asphalt mixtures. To investigate the effects of warm-mix agents on CRA, the viscosity values of all binders were measured at 135 °C, 145 °C, 150 °C, 165 °C, and 180 °C, as presented in Figure 3. It can be observed that the viscosity values of all binders decreased with increasing temperature. CRA exhibited higher viscosity, which indicates poorer flowability and workability. Compared with CRA, the viscosity values of CRA-2S and CRA-5U decreased, suggesting that Sasobit and UWM effectively reduce viscosity and improve the workability of CRA. Specifically, the viscosity of CRA-2S, CRA-5U, and CRA-2S5U at 135 °C declined by 11.9%, 24.1%, and 40.2%, respectively, compared with CRA, while at 180 °C, it declined by 9.7%, 21.8%, and 30.6%, respectively, for CRA-2S, CRA-5U, and CRA-2S5U. Moreover, the data were fitted using the Saal model, which has been widely validated in previous studies [35,36]. The fitting results are presented in Figure 4 and Table 7. According to JTG F40-2004, a viscosity below 3 Pa·s is regarded as the critical threshold for ensuring workability. Therefore, 3 Pa·s was selected as the target viscosity. Based on the fitting, at a target viscosity of 3 Pa·s, the mixing temperatures for CRA, CRA-2S, CRA-5U, and CRA-2S5U were 160 °C, 154 °C, 150 °C, and 146 °C, respectively. Moreover, in the Saal model, a higher fitting parameter *m* indicates greater temperature susceptibility of the binder. The *m*-value of CRA-2S was close to that of CRA, which suggests that the addition of Sasobit has a limited effect on the temperature susceptibility of CRA. In contrast, CRA-5U exhibits the highest *m*-value, which means that its viscosity decreases more rapidly with increasing temperature. The composite warm-mix agent also increased the temperature susceptibility of CRA. However, its effect was weaker than that of UWM, which indicates that the combination of Sasobit and UWM exhibits a balanced effect on the viscosity–temperature characteristics of CRA.

### 3.3. High-Temperature Rheological Properties

#### 3.3.1. Rutting Resistance Property

**Figure 5:**
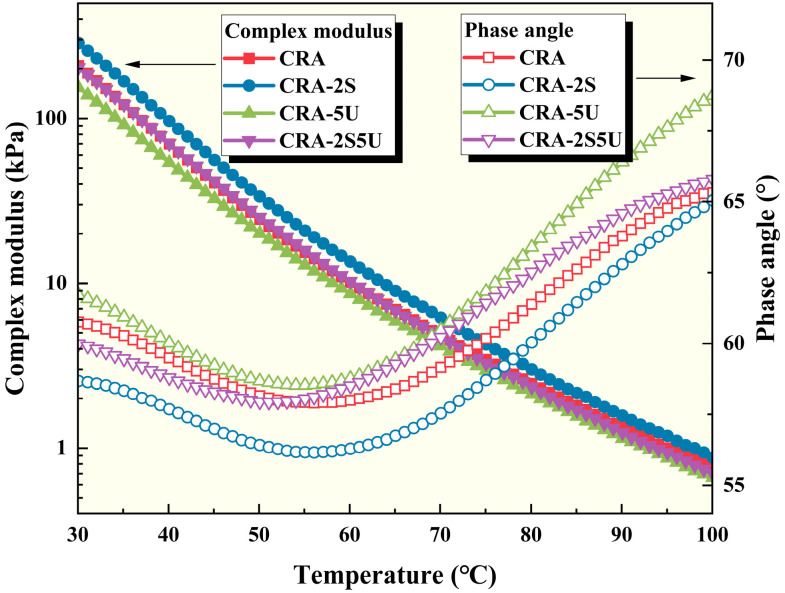
Complex moduli and phase angles of all binders.

Figure 5 illustrates the complex moduli (G*) and phase angles (δ) of all binders. It can be seen that the δ curves of all samples increase with increasing temperature, indicating that the viscoelastic behavior of CRA gradually transitioned from elasticity-dominated to viscosity-dominated. Compared with CRA, CRA-2S exhibited a lower phase angle, which suggests that the addition of Sasobit enhances the elastic component. In contrast, CRA-5U showed a higher phase angle than CRA, indicating that UWM increases the viscous component and reduces the elasticity of the binder. Moreover, the phase angle curve of CRA-2S5U lay between those of CRA-2S and CRA-5U. Notably, the addition of Sasobit intensified the stiffness of the binder, as reflected by the higher G*. Compared with CRA, the G* curve of CRA-5U shows a declining trend, while its δ curve rises, which indicates that UWM increases the viscous component of CRA and reduces its stiffness.

**Figure 6:**
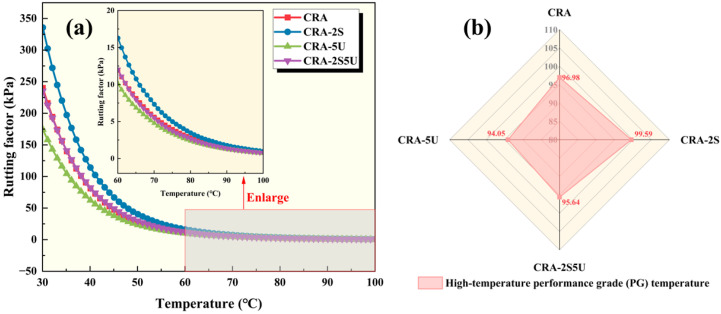
Rutting factor curves of all binders: (**a**) rutting factor curves and (**b**) high-temperature performance grade temperatures.

The rutting factor (G*/sin δ) was employed to evaluate the high-temperature rutting resistance of CRA, as shown in Figure 6a. A higher G/sin δ-value indicates stronger resistance. Across the entire temperature range, the G*/sin δ curve of CRA-2S is the highest, which indicates that it exhibits superior high-temperature rutting resistance, whereas that of CRA-5U shows the lowest, indicating that the addition of UWM diminishes the high-temperature rutting resistance of CRA. In contrast, the G*/sin δ curve of CRA-2S5U almost coincides with that of CRA, demonstrating that the composite warm-mix agent composed of Sasobit and UWM has a negligible impact on the high-temperature rutting resistance of CRA. In addition, the temperature at G*/sin δ = 1.0 kPa was defined as the high-temperature performance grade (PG) temperature according to SHAP, as shown in Figure 6b. The PG temperature increased by 2.6 °C for CRA-2S but decreased by 2.9 °C for CRA-5U compared with CRA, indicating that UWM deteriorates the high-temperature performance of CRA. The increase in the PG temperature for CRA-2S5U compared with CRA-5U demonstrates that the composite warm-mix agent alleviates this adverse effect.

#### 3.3.2. Creep and Recovery Property

**Figure 7:**
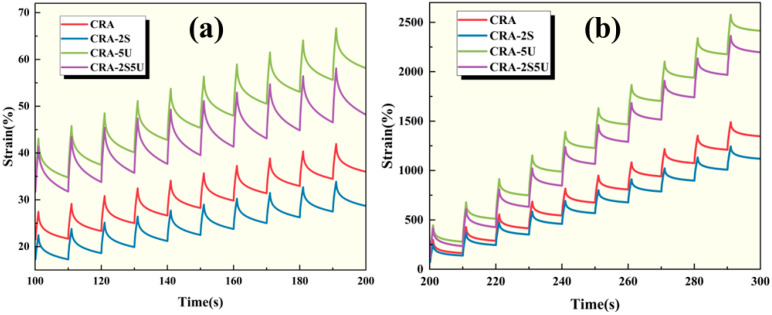
Strain curves of all binders obtained from the MSCR test: (**a**) 0.1 kPa and (**b**) 3.2 kPa.

Figure 7 displays the strain curves of all binders. The shear strain curve of CRA-2S is lower than that of CRA, indicating that the incorporation of Sasobit enhances the stiffness and high-temperature permanent deformation resistance of CRA. Conversely, CRA-5U exhibits significantly higher shear strain than CRA, which suggests that the addition of UWM weakens the stiffness of CRA and makes it more susceptible to cumulative deformation under cyclic loading.

**Figure 8:**
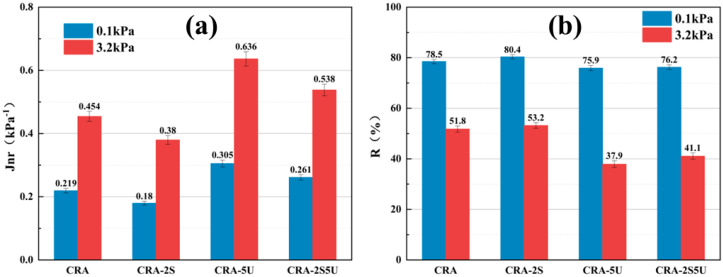
Percent recovery and non-recoverable creep compliance of all binders: (**a**) non-recoverable creep compliance and (**b**) percent recovery.

**Figure 9:**
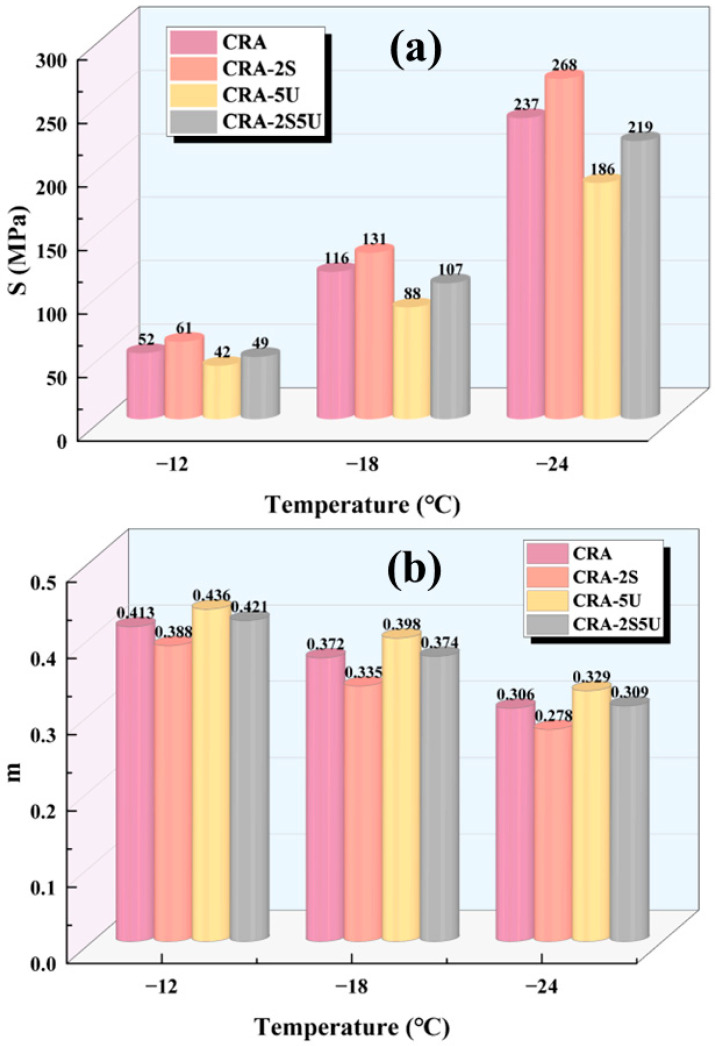
Low-temperature rheological parameters of all binders: (**a**) creep stiffness and (**b**) creep rate.

The high-temperature permanent deformation resistance of CRA was quantified using percent recovery (R) and non-recoverable creep compliance (Jnr), as illustrated in Figure 8. In general, a lower Jnr indicates less non-recoverable deformation after cyclic loading, whereas a higher R-value reflects a stronger elastic recovery of the binder upon unloading. It can be seen from Figure 9 that as the stress level rises from 0.1 kPa to 3.2 kPa, all binders exhibit an increased Jnr and a decreased R, indicating that higher stress levels significantly increase the non-recoverable deformation of CRA and diminish the elastic recovery ability of CRA. Moreover, compared with CRA, the R-value of CRA-2S at 3.2 kPa increases by 2.7%, whereas the R-value of CRA-5U decreases by 26.8%, and that of CRA-2S5U decreases by 20.6%, which indicates that the addition of UWM reduces the elastic recovery ability of CRA and is consistent with the DSR test results. In the DSR test, CRA-5U exhibits the highest phase angle curve, indicating the lowest elastic fraction in CRA, which limits its ability to store and recover elastic energy under applied stress and directly constrains its elastic recovery performance. In contrast, the phase angle curve of CRA-2S is the lowest, indicating the highest elastic fraction, which reduces plastic deformation and diminishes the accumulation of permanent strain. Compared with CRA-2S and CRA-5U, CRA-2S5U demonstrates a satisfactory elastic recovery ability, which suggests that the composite warm-mix agent mitigates the negative effect of UWM on the high-temperature permanent deformation resistance of CRA.

### 3.4. Low-Temperature Rheological Properties

The low-temperature rheological parameters (creep stiffness and creep rate) are displayed in Figure 9. As can be observed, with decreasing temperature, the creep stiffness of all binders increases steadily, while the creep rate (m-value) decreases, which indicates that the stress relaxation capacity of CRA is weakened and the risk of low-temperature cracking is intensified. According to the SHRP standards, the creep stiffness (S) should be ≤300 MPa, and the m-value should be ≥0.3. This criterion has been widely accepted for the evaluation of CRA [37,38]. All binders met the requirements at −12 °C and −18 °C. At −24 °C, the m-value of CRA-2S decreased to 0.278, whereas the other binders remained within the standard limits. The m-value of CRA-2S was lower than 0.3, which indicates that its applicability in cold regions is limited. In addition, at the same test temperature, the creep stiffness (S) of the binder samples followed the order CRA-2S > CRA > CRA-2S5U > CRA-5U, while the m-value decreased in the order CRA-5U > CRMA-2S5U > CRA > CRA-2S. These differences indicate that the incorporation of Sasobit markedly increases the stiffness of the binder while simultaneously reducing its stress relaxation capacity, which diminishes the low-temperature cracking resistance of CRA. Conversely, the addition of UWM decreases the stiffness of CRA while enhancing its flexibility. This improved flexibility allows the binder to maintain satisfactory bending resistance at low temperatures and mitigates the risk of cracking. The S-value of CRA-2S5U declined by 8.5%, and the m-value rose by 11.2%, which indicates that UWM can mitigate the adverse effect of Sasobit on the low-temperature performance of the binder. Therefore, compared with Sasobit, the composite warm-mix agent is more suitable for application in cold climates.

### 3.5. Fourier-Transform Infrared Spectroscopy Analysis

**Figure 10:**
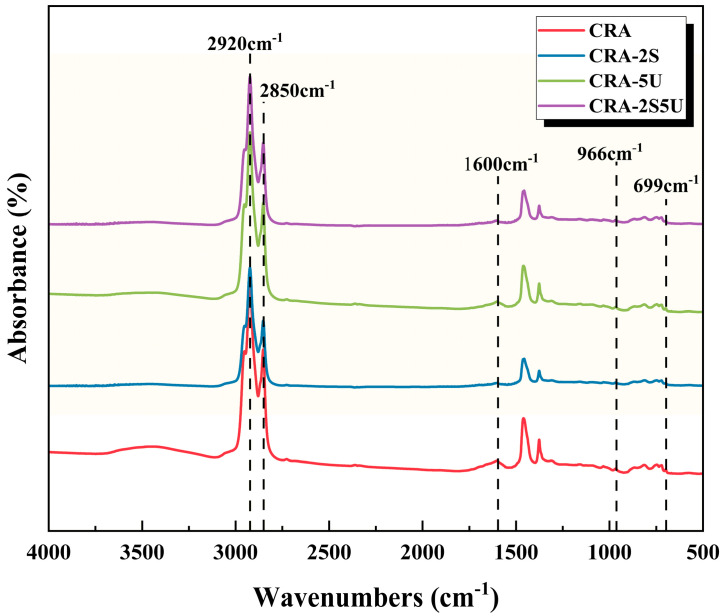
FTIR spectra of all binders.

The FTIR spectra of all binders are presented in Figure 10. The absorption peaks at 2850 cm^−1^ and 2920 cm^−1^ in the CRA are attributed to the stretching vibrations of aliphatic -CH_3_ and -CH_2_- groups, while the peaks at 1435 cm^−1^ and 1384 cm^−1^ correspond to the bending vibrations of aliphatic -CH_3_ and -CH_2_- groups. The absorption peak at 734 cm^−1^ is assigned to the vibration of methylene groups, which are characteristic of typical aliphatic structures. The absorption peak at 1600 cm^−1^ originates from the vibration of the aromatic ring skeleton and represents the characteristic aromatic structure of asphalt, while the intensity of this peak can be enhanced after incorporation of the CR. The absorption peak at 966 cm^−1^ is attributed to the rubber component of the CR, indicating the presence of the unsaturated butadiene structure in the rubber. The absorption peak at 699 cm^−1^ corresponds to the out-of-plane bending vibration of aromatic C-H in the aromatic ring of the CR. Moreover, it can be seen from the comparison between CRA, CRA-2S, CRA-5U, and CRA-2S5U that the addition of Sasobit and UWM does not result in the appearance of new absorption peaks or the disappearance of the existing peaks. This suggests that no chemical interaction occurs between Sasobit, UWM, and the CRA.

### 3.6. VOC Emission Analysis

**Figure 11:**
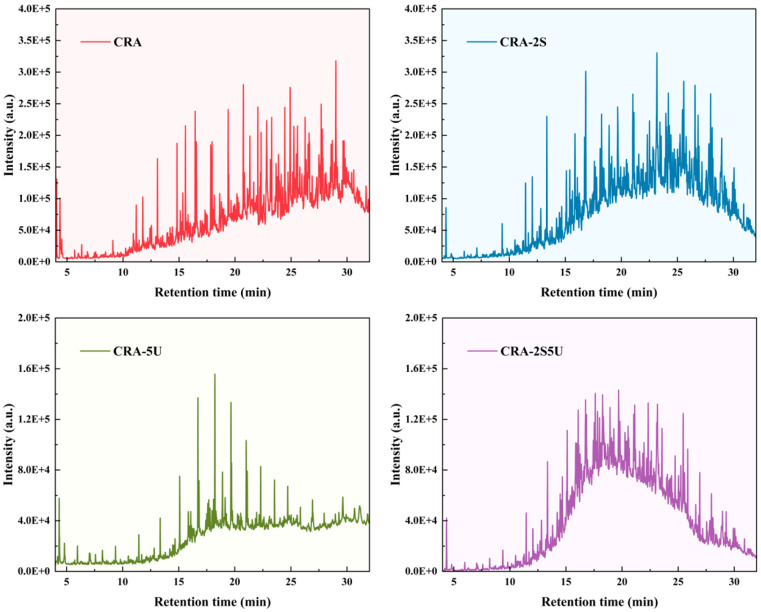
Total ion chromatograms (TICs) obtained from GC–MS test of all binders.

**Figure 12:**
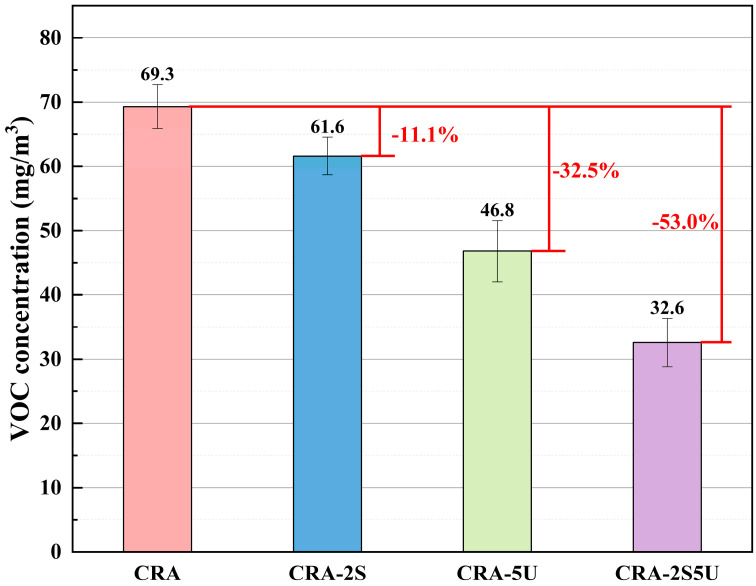
VOC concentrations of all binders.

**Figure 13:**
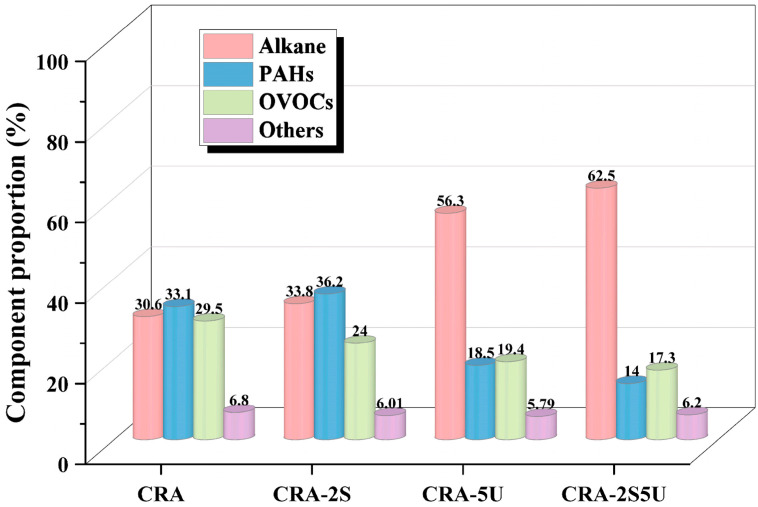
VOC component proportions of all binders.

The incorporation of warm-mix agents significantly reduces the viscosity and mixing temperature of CRA, which is accompanied by a decrease in VOC emissions. Accordingly, the temperature at which the binder viscosity reaches 3 Pa·s was selected as the VOC collection temperature based on the viscosity–temperature characteristics of all binders. The high viscosity of CRA requires excessively high heating to achieve the conventional mixing viscosity, which is difficult to achieve in the laboratory. The temperature corresponding to a viscosity of 3 Pa·s was adopted as the VOC collection temperature, and this selection is appropriate and safe for laboratory operation and reproducibility. Figure 11 presents the total ion chromatograms (TICs) of all binders. The VOC components of CRA, CRA-2S, CRA-5U, and CRA-2S5U are complex. The VOC emission concentration was calculated according to Equation (2), as shown in Figure 12. The VOC concentration of CRA reached 69.3 mg/m^3^, whereas that of CRA-2S decreased by 11.1%. The observed reduction in the VOC concentration is attributed to the incorporation of Sasobit, which leads to a lower mixing temperature and reduces VOC emissions from CRA. Compared with CRA, the VOC concentration of CRA-5U dropped by 32.5%, which is consistent with the viscosity–temperature characteristics discussed previously. Under the same viscosity condition, the mixing temperature of CRA-5U was lower than that of CRA-2S, which resulted in a further reduction in VOC emissions. Notably, compared with CRA, the VOC concentration of CRA-2S5U declined by 53.0%, which suggests that the composite warm-mix agent exhibits the most pronounced effectiveness. At the same viscosity level, CRA-2S5U showed the lowest mixing temperature, which directly led to the lowest VOC emissions. This result further confirms the viscosity-reducing effect of the warm-mix agent on CRA. Furthermore, given the constraints of the test method, the reported VOC concentration of the binders should be regarded as approximate rather than exact. However, the results remain adequate for evaluating the impact of warm-mix agents. The chemical components of VOCs were classified into alkanes, polycyclic aromatic hydrocarbons (PAHs), oxygenated volatile organic compounds (OVOCs), and others, as shown in Figure 13. In the CRA, alkanes account for 30.6%, while PAHs and OVOCs account for 33.1% and 29.5%, respectively, indicating that PAHs and OVOCs are the major components in the VOCs of the CRA. PAHs and OVOCs are generally considered hazardous to human health, particularly PAHs, which are recognized as carcinogenic substances. The addition of Sasobit only reduced the proportion of OVOCs. The incorporation of UWM significantly increased the proportion of alkanes and decreased the proportions of PAHs and OVOCs. These changes indicate that the UWM exhibits an inhibitory effect on emissions of PAHs and OVOCs from CRA, thereby reducing the potential health and carcinogenic risks.

In summary, an ideal warm-mix agent should exert minimal influence on the physical and rheological properties of CRA while effectively reducing its viscosity. This reduction in viscosity enables production, mixing, and paving at lower temperatures. Based on the aforementioned experimental results, Sasobit reduces the viscosity and mixing temperature of CRA and increases its stiffness, which enhances high-temperature rutting resistance and elastic recovery but compromises low-temperature cracking resistance. UWM also lowers the viscosity and mixing temperature of CRA while increasing its viscous fraction and flexibility. The elevated viscous fraction and enhanced flexibility are unfavorable for high-temperature permanent deformation resistance, yet they significantly mitigate the risk of low-temperature cracking. In contrast, the composite warm-mix agent consisting of Sasobit and UWM exhibits the most favorable warm-mix effectiveness while exerting the least impact on the physical and rheological properties of CRA, rendering it an optimal and well-balanced solution. Moreover, the cost analysis of 1 ton of different asphalt binders is presented in Table 8. The raw material dosages were determined according to the preparation schemes described above, while the additive was excluded from the major material cost calculation. The raw material dosages were determined according to the preparation procedures of different asphalt binders described above, while additives were excluded from the major material cost calculation. The material prices were based on local quotations in Fujian provided by Fujian Transportation Development High-Tech Co., Ltd. The cost of CRA-2S5U was 27.9%, 16.1%, and 7.6% higher than those of CRA, CRA-2S, and CRA-5U, respectively. However, the VOC concentration of CRA-2S5U was reduced by 53.0%, 47.1%, and 30.4% compared with those of CRA, CRA-2S, and CRA-5U, respectively. These results indicate that CRA-2S5U achieves substantial environmental benefit with relatively low additional economic cost, which is of great significance for protecting the health and safety of construction workers and nearby residents during the construction of CRA pavements.

## 4. Conclusions and Future Prospects

### 4.1. Conclusions

(1) Sasobit, as an organic warm-mix agent, can reduce the flowability of CRA and increase its elastic fraction, which enhances the stiffness of CRA. This effect also improves the resistance to permanent deformation of CRA, as evidenced by an increase in the high-temperature PG to 99.5 °C and a 16.3% reduction in the non-recoverable creep compliance at 3.2 kPa. However, it simultaneously compromises the low-temperature cracking resistance of CRA, as evidenced by a 13.1% increase in the −24 °C stiffness modulus.

(2) UWM, as a surfactant-based warm-mix agent, can increase the flowability of CRA and raise its viscous fraction, which contributes to a 21.5% increase in the −24 °C stiffness modulus of CRA. However, the increased viscous fraction weakens the high-temperature permanent deformation resistance of CRA, leading to a decrease in its high-temperature PG to 95.64 °C and a 40% increase in the non-recoverable creep compliance at 3.2 kPa.

(3) The composite warm-mix agent consisting of 2 wt.% Sasobit and 5 wt.% UWM can balance the viscoelastic fraction of CRA, which optimizes its stiffness and flexibility. This effect enables CRA to achieve satisfactory high-temperature rheological performance and low-temperature cracking resistance, while meeting engineering requirements. Moreover, Sasobit and UWM are mainly physically blended with CRA.

(4) Sasobit, UWM, and the composite warm-mix agent can all reduce the viscosity of CRA, which lowers the mixing, paving, and compaction temperatures as well as VOC emissions. The composite warm-mix agent exhibits the best warm-mix performance, reducing the VOC emissions and viscosity at 135 °C of CRA by 53.0% and 40.2%, respectively. Based on physical properties, rheological properties, and VOC emissions, the composite warm-mix agent demonstrates superior overall performance compared with Sasobit and UWM.

### 4.2. Future Prospects

This work investigated the effects of Sasobit and UWM on the physical properties, rheological properties, and VOC emissions of CRA. Due to space limitations, some aspects can be considered in future research.

(1) The effects of warm-mix agents on the aging performance of CRA should be systematically evaluated.

(2) The effects of warm-mix agents on the fatigue performance of CRA should be further investigated.

(3) The influence of warm-mix agents on the construction cost, energy consumption, and environmental benefit of CRA pavement throughout the whole life cycle should be investigated.

## Figures and Tables

**Table 1:** Main properties of the materials used.

Materials	Main Properties	
Base asphalt	Penetration (25 °C, dmm)	67.9
	Ductility (10 °C, cm)	30.9
	Softening point (°C)	48.1
	Viscosity (at 135 °C, Pa·s)	0.62
Crumb rubber	Ash content (%)	9.55
	Iron content (%)	0.03
	Density (kg/m^3^)	316
Aromatic oil	Density (20 °C, g/cm^3^)	0.99
	Flash point (°C)	220
	Viscosity (100 °C, mm^2^/s)	22
	Ash content (%)	0.01
Sasobit	Physical state	Milky white, solid
	Density (g/cm^3^)	0.940
	Flash point (°C)	>250
	Melting point (°C)	100–110 °C
	Chemical nature	Fischer–Tropsch wax
	Average molecular weight (g/mol)	1000
UWM	Physical state	Black, paste
	Density (g/cm^3^)	0.983
	Flash point (°C)	>260
	Melting point (°C)	——
Sulfur	Sulfur content	≥99.00
	Moisture content	≤1.00
	Ash content	≤0.20
	Sieve residue (>150 μm, %)	3
	Sieve residue (75–150 μm, %)	≤4.0

**Table 2:** Dosages of the warm-mix agents.

No.	Components	Abbreviation
1	2 wt.% Sasobit + CRA	CRA-2S
3	5 wt.% UWM + CRA	CRA-5U
4	2 wt.% Sasobit + 4 wt.% UWM + CRA	CRA-2S4U
5	2 wt.% Sasobit + 5 wt.% UWM + CRA	CRA-2S5U
6	2 wt.% Sasobit + 6 wt.% UWM + CRA	CRA-2S6U

**Table 3:** Details of the temperature sweep test (TS).

Test	Temperature	Strain	Plate	Gap	Heating Rate	Stress
TS test	30~100 °C	1%	25 mm	1 mm	1 °C/min	—
MSCR test	64 °C	—	25 mm	1 mm	—	0.1 kPa, 3.2 kPa

**Table 4:** Details of the gas chromatography (GC) test.

Parameter	Split Ratio	Sample Preheating	Temperature Ramp	Carrier Gas	Carrier Gas Flow Rate
Settings	40:1	72 °C for 4 min	8 °C/min to 280 °C, held for 2 min	Helium	1.2 mL/min

**Table 5:** Details of the mass spectrometry (MS) test.

Parameters	Ionization Mode	Ion Source Temperature	Solvent Delay	Capillary Column	Mode
Settings	Electron ionization (70 eV)	230 °C	3 min	DB-5MS (30 m × 0.25 mm × 0.25 μm)	Full-scan mode (*m*/*z* 45–550)

**Table 6:** Relationship between toluene standard concentration and chromatographic peak area.

Toluene Concentration (mg/m^3^)	Chromatographic Peak Area	Linear Fitting Result
5	1,187,564	Y = 245,097X + 101,250, R^2^ = 0.9995
10	2,275,123	
50	12,475,655	
100	24,751,234	
150	36,622,133	

**Table 7:** Fitting results using the Saal equation.

Types	Fitting Equations	R^2^
CRA	lglg(η*1000) = 6.2526 − 2.1675lg(T + 273.13)	0.9871
CRA-2S	lglg(η*1000) = 6.2585 − 2.1729lg(T + 273.13)	0.994
CRA-5U	lglg(η*1000) = 6.6127 − 2.3111lg(T + 273.13)	0.9892
CRA-2S5U	lglg(η*1000) = 6.5284 − 2.2829lg(T + 273.13)	0.9764

**Table 8:** Cost analysis of different asphalt binders (per ton).

Raw Materials	Unit Price (CNY/t)	Raw Material Dosage for 1 Ton of Asphalt (t)			
		CRA	CRA-2S	CRA-5U	CRA-2S5U
70# petroleum asphalt (Base asphalt)	3500	0.741	0.730	0.714	0.704
Crumb rubber	2000	0.222	0.219	0.214	0.212
Aromatic oil	4200	0.037	0.036	0.036	0.035
UWM	20,000	——	——	0.036	0.035
Sasobit	25,000	——	0.015	——	0.014
Total cost (CNY/t)	——	3192.9	3519.2	3798.2	4085

## Data Availability

The original contributions presented in this study are included in the article. Further inquiries can be directed to the corresponding author.
